# Gravity Stress Radiograph Revealing Instability at the First Metatarso-Cuneiform Joint in Lisfranc Injury

**DOI:** 10.7759/cureus.1015

**Published:** 2017-02-07

**Authors:** Pejma Shazadeh Safavi, William Weiss, Vinod Panchbhavi

**Affiliations:** 1 University of Texas Medical Branch at Galveston; 2 Department of Orthopedic Surgery & Rehabilitation, Texas Tech University Health Sciences Center Paul L Foster School of Medicine; 3 Department of Orthopedics, University of Texas Medical Branch at Galveston

**Keywords:** trauma, digital x-ray & casting, foot ankle

## Abstract

Lisfranc injuries are commonly missed in the acute setting, largely due to subtle findings that often require weightbearing radiographs or more complex imaging for diagnosis. The long-term consequences of missed injuries are debilitating osteoarthritis of the midfoot, but this may be prevented with appropriate diagnosis and treatment. This case study presents a Lisfranc injury initially diagnosed by gravity stress radiograph. While used for other injuries, there is no literature on the use of gravity stress radiographs for diagnosis of Lisfranc injuries. The use of this simple technique to accurately diagnose Lisfranc injuries may improve detection and patient outcomes.

## Introduction

The Lisfranc ligament which connects the medial cuneiform to the second metatarsal base is one of the major stabilizing structures of the tarsal-metatarsal joint. Injuries to the Lisfranc ligament are common in high energy motor vehicle accidents and in low energy sports injuries when the foot is twisted while bearing weight [1,7]. Despite this important role in stabilizing the foot and its transverse arch, injuries to the Lisfranc ligament are commonly misdiagnosed. Signs of a Lisfranc injury may be subtle or absent on both physical examination and initial radiographs, contributing to clinical error [1-2,6-7].

Radiographic signs of Lisfranc injury and instability include subluxation of the tarsal-metatarsal joint on the lateral view and diastasis at the base of the second metatarsal and the medial cuneiform on an anteroposterior (AP) view [3,5-7]. However, non-weightbearing radiographs may not reveal subtle instability of the Lisfranc joint, which can only otherwise be seen with stress views or advanced imaging [4,6]. The literature indicates that up to 20% of these injuries may be misdiagnosed or missed [1,9]. If injuries to the Lisfranc ligament are unrecognized, the joints of the midfoot are subjected to abnormal stresses and undergo degenerative changes, resulting in arthritis [1,4-7].

In acute Lisfranc injuries, weightbearing radiographs or manipulation under anesthesia with fluoroscopy can reveal abnormal dynamic instability in the midfoot [8]. Non-weightbearing radiographs may fail to reveal subtle injuries as destabilizing stress is not imposed on the region of interest, but gravity stress radiographs utilize gravity to impose such dynamic stress on the foot which may unmask more subtle injuries. Intraoperatively, dorsal capsular disruption is also often evident in the Lisfranc joint. The purpose of this study is to present the case of a patient with Lisfranc injury exhibiting instability at the first metatarso-cuneiform joint identified by lateral gravity stress radiograph. Weightbearing radiographs obscured the injury by maintaining reduction of the instability. A lateral gravity stress radiograph may have potential to reveal instability at the first metatarso-cuneiform joint in acute Lisfranc injuries and may be of diagnostic utility.

## Case presentation

A 64-year-old male presented to the orthopedic clinic with foot pain following a moped accident. He did not recall the exact mechanism of injury but did remember planting the affected foot on the ground before falling off of the moped. He was seen in an emergency department at the time of the accident, cleared of significant injury and placed in a walking boot for pain control. His chief complaint on presentation to the clinic was continued left foot pain, localized to the midfoot and great toe. There were no other complaints and there was no history of injury to the affected extremity. He is otherwise healthy, with no significant medical history. Physical exam was significant for ecchymosis and edema of the left midfoot, including plantar ecchymosis. The neurovascular examination was intact and tenderness to palpation was noted at the first tarsal-metatarsal joint. No other abnormalities were identified.

**Figure 1:**
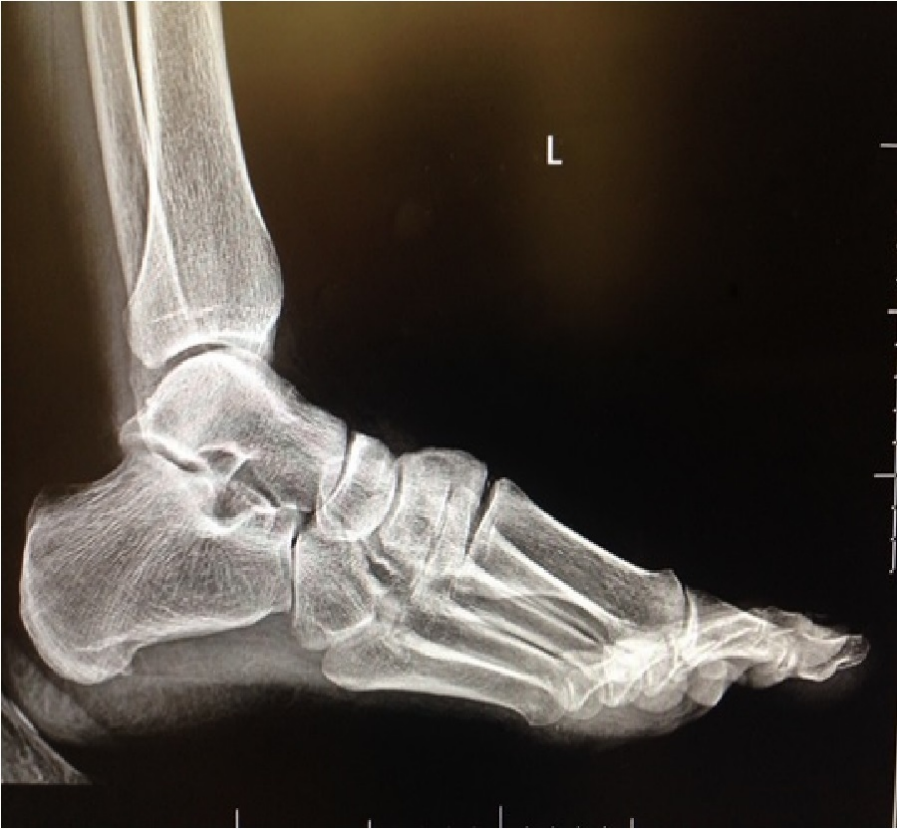
Preoperative gravity stress lateral radiograph of the left foot demonstrating widening of the first metatarsal cuneiform joint due to dorsal capsular disruption

**Figure 2:**
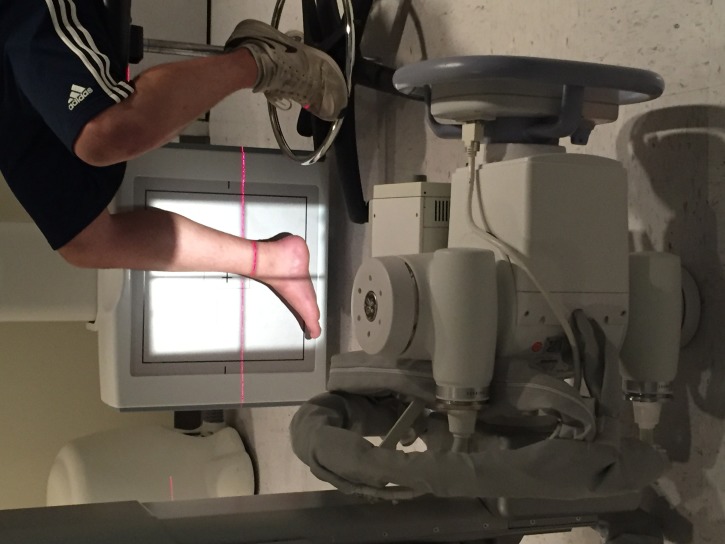
Gravity stress view technique demonstration

**Figure 3:**
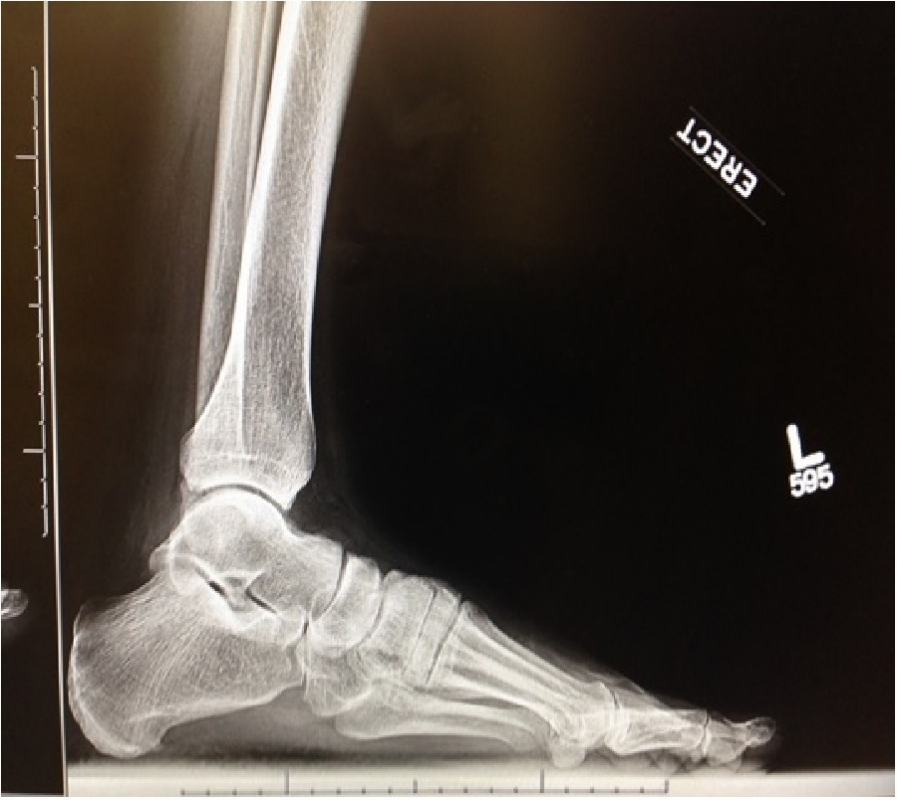
Preoperative weight bearing lateral radiograph of the left foot demonstrating reduction of the first metatarsal cuneiform joint despite dorsal capsular disruption and instability of the joint

**Figure 4:**
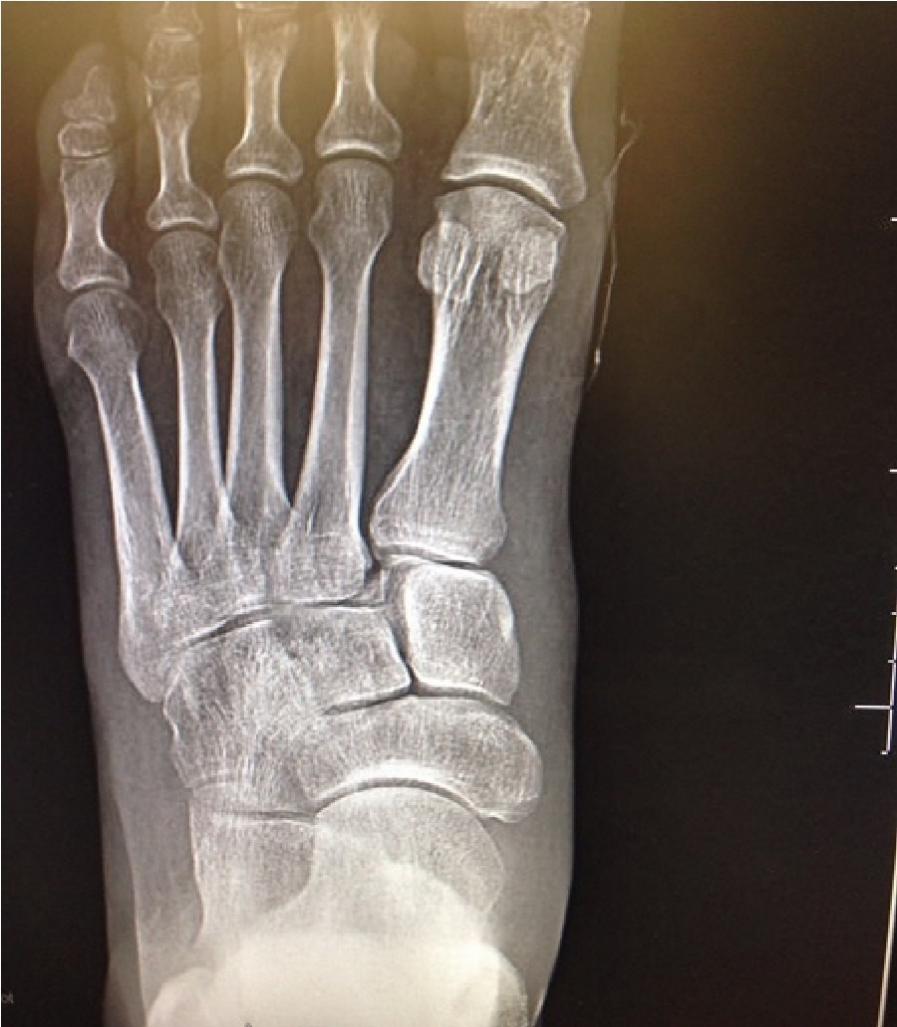
Preoperative anteroposterior (AP) view of the left foot demonstrating non-displaced fracture of the proximal phalanx of the first toe and subluxation with fracture of the base of the second metatarsal (‘fleck sign’) indicative of Lisfranc injury

 A non-weightbearing (gravity stress) lateral radiograph of the affected foot taken at the time of injury demonstrated significant dorsal widening of the first tarsal-metatarsal joint (figure 1). Figure 2 demonstrates the technique used to take this radiograph. Subsequent weightbearing views taken in clinic revealed oblique intra-articular fracture of the great toe proximal phalanx but reduction of the dorsal opening seen in non-weightbearing lateral radiograph (figure 3 and figure 4).

**Figure 5:**
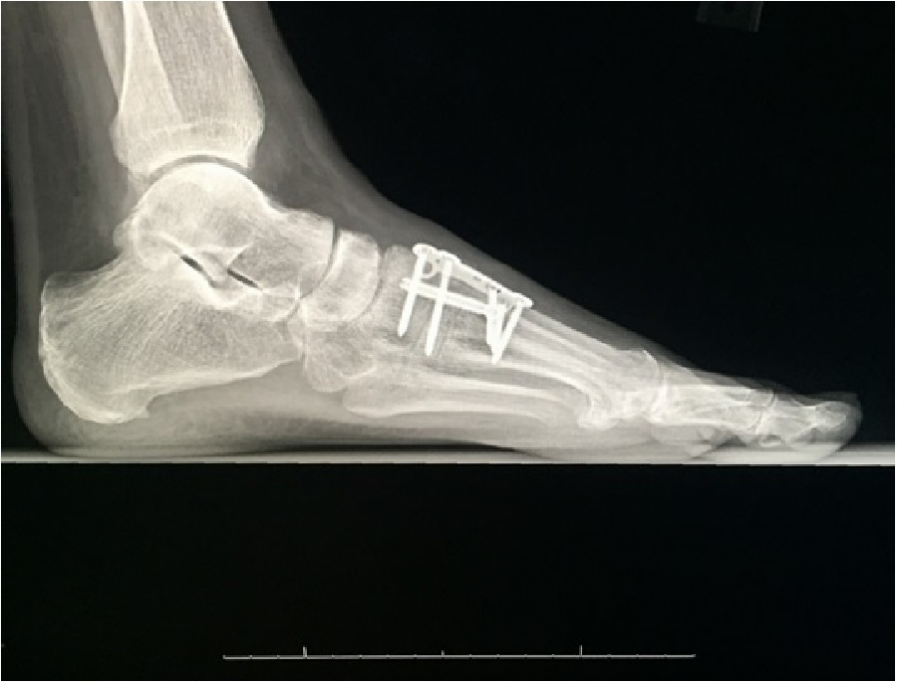
Postoperative weightbearing lateral radiograph of the left foot demonstrating joint-sparing stabilization of the first metatarsal cuneiform joint and Lisfranc joint fixation with a screw

**Figure 6:**
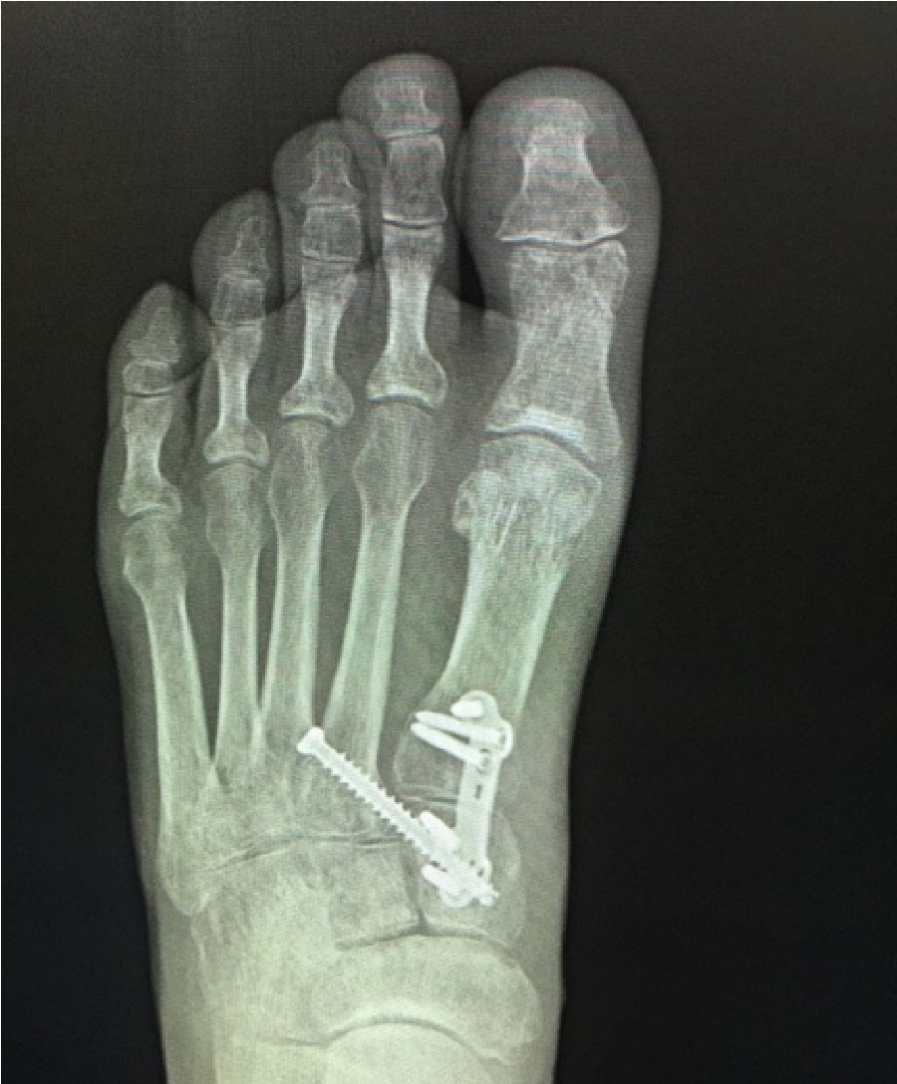
Postoperative weightbearing anteroposterior (AP) view of the left foot demonstrating reduction and jointsparing fixation of the first metatarsal cuneiform joint and fixation of the Lisfranc joint with a screw

Based on this information, the patient was diagnosed with an unstable Lisfranc injury and underwent operative reduction and joint-sparing fixation of first metatarso-cuneiform joint and placement of a screw across the Lisfranc joint (figure 5 and figure 6). Intraoperatively, disruption of the dorsal capsule at the first metatarso-cuneiform joint was observed and instability of the Lisfranc joint was demonstrated. The postoperative course was without complications and the patient was kept non-weightbearing on the operative extremity for three months. The patient progressed as expected and is now weightbearing as tolerated on the affected foot with stability of the Lisfranc joint maintained at last follow-up.

## Discussion

Lisfranc injuries are commonly misdiagnosed, resulting in inadequate treatment and debilitating degenerative arthritis [1,4-7]. In the present case study, a gravity stress lateral radiograph effectively identified an unstable Lisfranc injury that was obscured by weightbearing views. Simple and effective radiographic modalities for the diagnosis of Lisfranc injuries may improve both identification and outcomes for these patients.

Gravity stress radiographs for the diagnosis of Lisfranc injury have not been described in the literature. In cases where uncertainty exists regarding the presence or degree of instability at the Lisfranc joint, a weightbearing radiograph or stress examination under anesthesia with fluoroscopy are commonly used for definitive diagnosis [8]. Magnetic resonance imaging (MRI) or computed tomography (CT) scan may also be useful but are often a challenge to interpret acutely. However, patient discomfort, cost, and time involved in these examinations can be significant. Gravity stress radiographs are used for suspected ligamentous injuries of joints such as the ankle joint, and are simple and effective in diagnosing instability [9]. These views may be better tolerated by the patient after acute trauma when compared to physical stresses, while still providing useful diagnostic information [9]. In this case study of a Lisfranc injury, a gravity stress view revealed disruption of the dorsal capsule obscured by weightbearing views and assisted in the diagnosis of this often missed injury.

Limitations of this investigation include those inherent in its design as a case study of a singular patient and injury. In addition, while standard radiographic views did not identify this injury, more advanced imaging (MRI, CT) may have been able to do so. The pattern of ligamentous disruption in Lisfranc injuries is a continuum, believed to progress from dorsal to plantar with increasing injury severity. The instability identified by gravity stress view is presumably due to dorsal-capsular disruption. While this is thought to be the first area injured, this may not be so in every injury, making the gravity stress view less useful in these instances. Further study of both the progression of Lisfranc injuries and the utility of gravity stress views is warranted.

## Conclusions

This case report demonstrates the use of gravity stress radiographs in the diagnosis of Lisfranc injuries including dorsal capsular disruption. The use of gravity stress radiographs in place of other existing techniques may improve diagnostic accuracy for these injuries which have significant complications if missed.
